# Finger Angle Estimation From Array EMG System Using Linear Regression Model With Independent Component Analysis

**DOI:** 10.3389/fnbot.2019.00075

**Published:** 2019-09-26

**Authors:** Sorawit Stapornchaisit, Yeongdae Kim, Atsushi Takagi, Natsue Yoshimura, Yasuharu Koike

**Affiliations:** ^1^Department of Information Processing, Tokyo Institute of Technology, Tokyo, Japan; ^2^Institute of Innovative Research, Tokyo Institute of Technology, Yokohama, Japan; ^3^PRESTO, JST, Saitama, Japan

**Keywords:** array EMG system, multichannel surface EMG, musculoskeletal models, surface ElectroMyoGraphy (EMG), adaptive mixture ICA (AMICA), finger motion, convolutional pose machines

## Abstract

Surface ElectroMyoGraphy (EMG) signals from the forearm used in prosthetic hand and finger control systems require precise anatomy data of finger muscles that are small and located deep within the forearm. The main problem of this method is that the signal quality depends on the placement of EMG sensor, which can significantly affects the accuracy and precision to estimate joint angles or forces. Moreover, in case of amputees, the location of finger muscles is unknown and needed to be identified manually for EMG recording. As a result, most modern prosthetic hands utilize limited number of muscles with pattern recognition to control finger according to pre-defined grip which is unable to mimic natural finger motion. To address such issue, we used array EMG sensors to obtain EMG signals from all possible positions on the forearm and applied regression method to produce natural finger motion. The signals were analyzed using independent component analysis (ICA) to find the best-fitted independent component (IC) that matches the anatomical data taken after the experiment. Next, from the IC and EMG signals, finger angles were estimated using linear regression model (LRM). Each finger was assigned EMG and IC component for flexion and extension muscles, to assess the possibility of controlling each finger angle separately. We compared the joint angles of each finger between calculated from IC and EMG by correlation coefficients (CC) for all fingers. The average CC values were higher than 0.7, demonstrating the strength of the linear relationship. The different between IC and EMG methods suggests that the IC method can reduce noise and increase the signal to noise ratio. The performance of ICA method showed higher CC value at around 0.2 ± 0.10. In order to confirm the performance of ICA method, we also tested mathematical musculoskeletal model (MSM). The result from this study showed that not only array EMG sensors with ICA significantly improve the quality of signal detected from forearm but also reduce problems of conventional EMG sensors and consequently improve the performance of regression method to imitate natural finger motion.

## 1. Introduction

The human hand consists of five fingers to create intricate primary tools that we use to interact with the external world. Thanks to rapid advances in engineering and biotechnology, prosthetic hands have shown high accuracy in finger control with force adjustment that can mimic human finger motions. In order to capture the human intent to control finger, surface ElectroMyoGraphy (EMG) was used in many researches (Khushaba and Kodagoda, 2012; Zhang et al., 2014; Haris et al., 2015; Bai et al., 2016; Kawano and Koganezawa, 2016; Sahoo et al., 2016). The EMG signal was rectified, and low-pass filtered to reduce noise. Then, various models were used to extract hand postures from the processed EMG signal. Recently, one group succeeded to control fingers and wrist of a robot with predetermined grips so that the fingers and wrist angles of robot matched with the hand posture (Hargrove et al., 2013). However, such method did not reproduce the actual finger control or show agility to perform rapid motion. A previous research showed the possibility to convert EMG signal into muscle contraction force for direct control of the prosthetic device (Kawase et al., 2012). EMG signals of amputees were also used successfully to control a prosthetic device (Haris et al., 2015). However, such methods also encounter the problem of sensor location that affects the signal quality and consequently the performance of the algorithm that converts bio-signals to prosthetic motions.

Knowledge of the muscular and skeletal anatomy is crucial to identify exact muscle locations. Normally, the placement of the EMG sensor is based on experience and trial-and-error (Mercer et al., 2006). Without knowing exact location of the muscles, only large muscles close to the surface tend to be selected for use. This reduces the possible target muscles available to control prosthetic devices, leaving only large flexion-extension muscle pairs in the shoulder, elbow, and wrist. The small and deeply-located finger muscles are hard to detect and provide low signal quality for classifying motions to control the devices. As a result, most finger motion classifications use small number of muscles to control a limited predefined finger poses. Moreover, the finger muscles location may change depending on relative angle between elbow and wrist, which make EMG detection of a particular finger muscle more difficult.

Array EMG systems have been used to record single muscle fiber action potentials to investigate properties and firing patterns of motor units (MUs) (Zwarts and Stegeman, 2003). Using such devices with High-density (HD) surface electromyogram grids, we can analyze fatigue of the muscle fiber (Cifrek et al., 2009). Due to advances in signal processing, there are multiple methods to analyze signals, whose source are hidden or unknown. The best example is electroencephalography (EEG) analysis, where multiple electrodes are placed around the head to detect signals from neurons that are located deep within the skull. EEG is a method to detect electrical impulses that occur in the brain when brain cells communicate with each other (Hjorth, 1970). Generally, EEG is used to identify problems in the electrical activity of the brain to diagnose brain disorder such as seizures and head injuries.

Independent Component Analysis (ICA) is a signal processing method to separate independent signals that are linearly mixed in multiple sensors. This method is used in EEG analysis to reduce noise or remove artifact unrelated to the task such as blinks, head motion, and signals from facial muscles (Delorme et al., 2007). The musculoskeletal model (MSM) is the 2-order linear regression which represented by one degree of freedom joint for flexion and extension to reduce the complexity of finger musculoskeletal structure (Shin et al., 2009). Musculoskeletal models (MSM) required a smaller number of variables and train data. The variable of MSM will be acquired by finding minimal error using characteristic of each finger muscles according to the proposed model (Kawase et al., 2012). Linear regression model (LRM) was used to predict direction of movement of supination and pronation (Hahne et al., 2014). In order to reached out to researcher who might not be familiar with MSM, LRM were used to predict five finger angle in this study.

This study adapted electroencephalography (EEG) analysis with EMG measurement to achieve better signal quality from deep muscles. We built arm mask in consideration of hand anatomy and experimentation on several method to achieve high quality signal. The motivation to build such a system came from effort to standardize EMG signal measurement and to reduce the affect from operator personal skill. Moreover, we used the proposed system to overcome (1) anatomy variation of each subject, (2) disruption of EMG signal caused by adjacent joint movement, and (3) noise from skin movement.

In this paper, we proposed a new method using ICA and array EMG system around the forearm. The EMG signals were extracted from selected areas using anatomical data. The IC signals estimated from the EMG signal using ICA were plotted to confirm that the selected IC represents finger muscles. Finally, the estimated finger angle and measured finger angle were compared using correlation coefficients (CC).

## 2. Materials and Methods

### 2.1. Subjects

Ten healthy, right-handed, human participants (7 males, 3 females), between 24 and 28 years of age (mean 26.2 ± 1.8), participated in this study. The study protocol was approved by the ethics committee of the Tokyo Institute of Technology and was carried out in accordance with the Declaration of Helsinki. Written informed consent was obtained from each participant before the experiment.

### 2.2. Experiment Protocol

**Figure 1:**
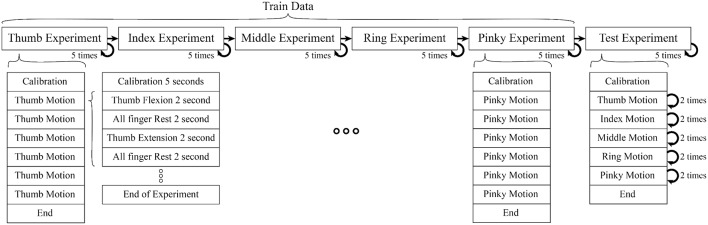
Each motion composed of 2 s period of finger flexion, rest, extension, rest (sum up to 8 s per motion), One experiment consisted of 6 motions. In train data, each experiment had isolate finger movement but in test data combine finger movement were performed (Thumb, Index, Middle, Ring, Pinky). In each experiment we also included calibration period to record data for initial position of finger, offset voltage, and noise level.

Each subject performed 25 experiments (5 experiments per finger without interruption, in the order of the Thumb, Index, Middle, Ring, and Pinky) to train the classifier. After theses 25 experiments, further 5 experiments were performed where all fingers were moved one after another in each experiment to verify our classifier's performance (see Figure 1). In each experiment, the calibration period was set before any motion to reduce noise. Each motion consisted of rest, flexion, and extension motions.

The experiment was separated into two parts (1) Training motion to estimate the parameters of the muscles model (Kawase et al., 2012) (2) Testing motions to verify the proposed method. The experiment was set up to minimize the interference between different finger motions to acquire a better EMG signal.

**Figure 2:**
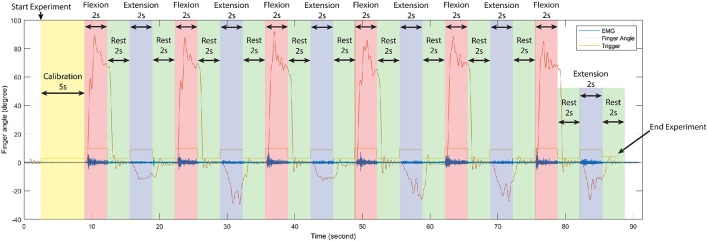
Time serial data of the first cycle of train experiment from a single subject, separate each period in one experiment according to stimulus signal (trigger) to observe changing between finger angle (thumb), and EMG signal (sensor channel 2). In each train experiment of our experiments were conduct in the same manner.

During train experiment, subjects flexed finger to its maximum range motion (around 0.9 ± 0.175 rad or 162 ± 10°) for 2 s, rested for 2 s, extended the same finger to its maximum range motion (around −0.20 ± 0.175 rad or −36 ± 10°) for 2 s, and rested for another 2 s. This cycle was repeated 6 times per experiment. Figure 2 shows the first cycle of a sample train experiment from a single subject. Likewise, in each test experiment the flexion-extension-rest cycle was repeated twice per finger, for a total 10 cycles.

Due to the interconnection between finger muscles, no subject can control each finger in total isolation. When attempting to move the ring finger, it will likely cause movements in the middle and pinky fingers. However since our aim was to study the natural motion of the human finger, the subjects were instructed to move a finger without attempting to resist the motion of other fingers, which should be as relaxed as possible.

### 2.3. Independent Component (IC) Analysis

Independent Component Analysis separates independent sources linearly mixed in several sensors. As we assumed that the signal from the muscles should be scattered to many sensors around the forearm, this method was used to extract the EMG signal of muscle groups that responded to flexion and extension of specific fingers.

EMG data were loaded into MATLAB using MoBILAB toolbox51 and exported to the EEGLAB version 15 (https://sccn.ucsd.edu/wiki/EEGLAB) to perform the following preprocessing: re-sampling, band-pass filtering between 5 and 200 Hz and the data were resampled to 500 Hz to reduce computational time. Among several ICA algorithms in EEGLAB, we used adaptive component analysis (AMICA) due to following features (Palmer et al., 2012):

- Adaptive Source Densities: AMICA uses a mixture of Generalized Gaussian density model to estimate source density which results in an extremely good fit between the density model and the actual density of the source is estimated.
- Multiple/Mixture Models: AMICA allows multiple ICA models to be learned simultaneously, automatically segmenting the data into regions of local stationarity, and returning a set of components for each model. AMICA can also be set to share components between models to increase estimation efficiency.
- Data Likelihood (Model Probability): joint probability distribution of each model is used to allowing rejection of unlikely data, as well as classification of new data.
- Parallel Implementation: program can use multiple cores in single workstation (using portable OpenMP code), as well as multiple nodes in a cluster (using portable MPI code). All binaries allow multi-core (SMP) execution. Only the Linux version currently supports clusters (we use the freely available Rocks / Sun Grid Engine).

All calculation was perform in middle to high end computer with specification:

- OS Name Microsoft Windows 10 Pro System Type x64-based PC.
- Processor Intel(R) Xeon(R) CPU E5-1680 v4 @ 3.40GHz, 3401 Mhz, 8 Core(s), 16 Logical Processor(s).
- Installed Physical Memory (RAM) 64.0 GB.
- Intel(R) C610 series/X99 chipset.
- NVIDIA Quadro P2000.

Also AMICA has a capability to learn a mixture of ICA models including but not limit to the ones related to muscles activity.

**Figure 3:**
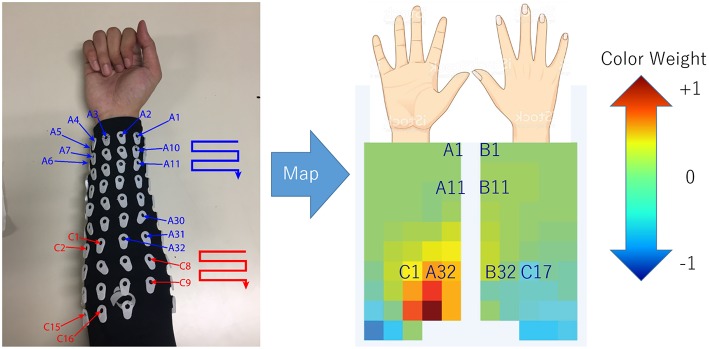
Configuration (July 2018) of array EMG system with topology plot display IC weight as color from blue to red. This plot was invented to investigate the relation between weight of each channel in minimal time.

**Figure 4:**
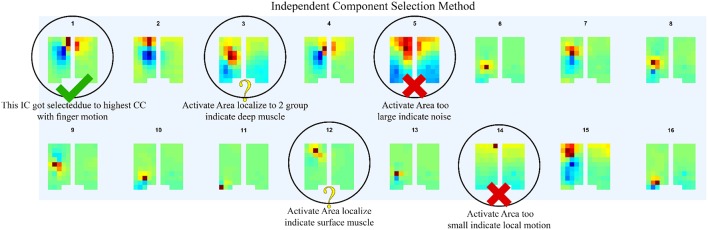
After compare normalize filtered EMG signal with finger motion, 16 highest CC values was displayed along with its topology plot to remove some component which likely be motion noise (large area of signal), sensor detach (signal from one sensor with large reverse sensor), and co-contraction of other muscles (anatomical data that do not match with motion).

In order to determine the source of the IC signal, we compared its location with the anatomical data. We converted the weight of each IC into a geometry or topology plot, where the color indicates the weight a particular IC assigned to each sensor in the EMG array as shown in Figure 3. the CC between every IC component with finger flexion and finger extension were calculated. The IC with the largest 16 CC values was displayed along with its topology plot to remove motion noise in order to find the best IC from the location that corresponds to the anatomical data (see Figure 4).

### 2.4. Topology Plot of IC Weight

The topology plot shows the weight of the IC from sensors, which were separated into two parts that represent flexion (inner arm) and extension (outer arm) muscle groups, as shown in Figure 3.

Flexion muscle group consisted of A1–A32 and C1–C16. Likewise, the extension muscle group consisted of B1–B32 and C17–C32. The sensors were arranged in a zigzag pattern due to the configuration of bio-semi sensor which group sensors into 4 sensors per group and limit the distance between the sensors. In order to simplify the sensor placement, the image of the topology plot was used to replace the sensor channel name and showed the relative geometry location of sensors on forearm. From here onwards, we described the array EMG system in terms of the topology plot rather than the sensor channel labels.

**Figure 5:**
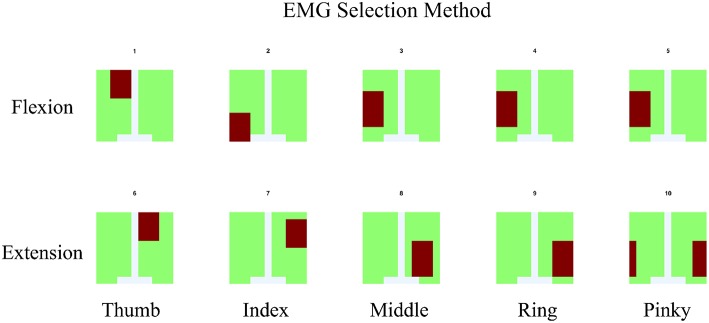
For EMG signal, the average of signal from area which co-response to anatomical finger muscles location was used. The area was indicated by red color.

The EMG signals acquired from the device were separated according to the anatomical data of the subject. The general concept of muscle locations according to finger and motion of the finger were shown (see Figure 5). The signal of each monopole EMG signal was compared with the finger motion to find the electrode that provided the best-fitted EMG signal for a specific finger's flexion and extension.

### 2.5. Realsense With Convolutional-Pose-Machines Finger Tracking

**Figure 6:**
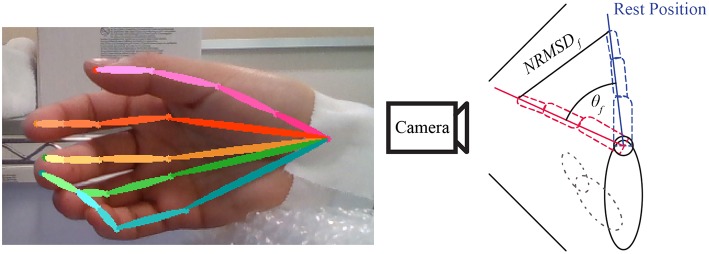
Example of convolutional-pose-machines by tensorflow in the preliminary experiment. The finger angle is the normalized root mean square error (NRMSE) of finger 2D position detected by convolutional-pose-machines. The direction of finger motion (forward or backward from camera) is indicated by stimulus or trigger signal.

To have a ground-truth measurement of the finger angles, we used the convolutional-pose-machines by tensorflow (Wei et al., 2016), a state-of-the-art pose estimator, to detect the finger position in 2D space (see Figure 6). The 21 joints' 2D position from the five fingers were fed into a hand-finger-model, which was trained using example images from a Realsense depth camera. The default lengths of each finger were recorded in the calibration period of each experiment (see Figure 2). A low-pass filter with a cutoff frequency of 1 Hz was applied to the data to reduce noise and error during pose estimation.

Finger angle (θ_*f*_) is composition of Trigger (*T*_*dir*_) provide direction of flexion and extension with Normalized root-mean-square error (NRMSE) as shown in Equation 1. The value of Trigger is depended on experiment trigger value, flexion motion is 2.094 radian or 120 degree and extension motion is −0.523 radian or −30 degree.

(1) $$\begin{array}{r} \theta_{f}=T_{dir}\cdot NRMSD_{f} \end{array}$$

Finally, low-pass and median filters were applied to reduce noise in the data from both the camera and the estimated finger positions from the convolutional-pose-machines. In some subject, adjustment between every rest period was required because the subjects moved their hand in unexpected direction and the camera cannot capture the finger motion. The motion data was recorded throughout the experiment to confirm that convolutional-pose-machines were able to detect the fingers at all time. If not, researcher would ask the subject to redo that part of experiment.

To confirm the performance of the Realsense with convolutional-pose-machine's finger tracking, we used conventional motion tracking using an OptiTrack with Baseline Upper Body + Fingers (33). However, this system could only track three fingers (Thumb, Index, and Pinky) at a time. Thus, the Thumb, Index and Pinky fingers were used to determine the match in the finger angle between the convolutional-pose-machine and the Optitrack system. Their CC were high at 0.9040 ± 0.1, implying that the convolution-pose-machine provides an accurate ground-truth measurement of the finger angles.

### 2.6. EMG Data Acquisition

Participants sat on the chair and were given instructions from a screen placed 0.5 m in front of them. Subject performed the finger motion in front of the Realsense camera that captured their motion. The array EMG system was attached to the right forearm of the participants.

**Figure 7:**
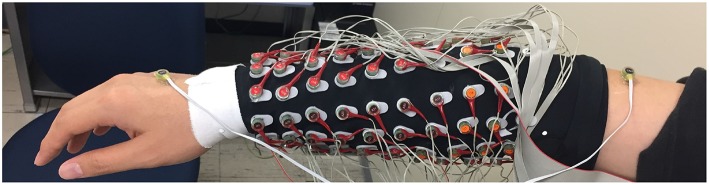
General position of Bio-semi electrode sensor in all experiment, The active and passive reference electrodes (CMS and DRL electrodes) placed between the hand and upper arm. However, in subjects that the distance between the hand and upper arm are too far part for CMS and DRL electrodes to activate, the CMS and DRL electrodes were placed on the back of the subject hand 4 cm apart. This alternative method might increase noise but unavoidable.

The experimental program was created using MATLAB 2015b (The MathWorks, Inc., U.S.A.), and the visual stimuli were presented on a 19-inch LCD. We acquired EMG using a Biosemi Active Two amplifier system with active sensors (Biosemi, Amsterdam, Netherlands). EMG signals were recorded from 96 positions with 24-bit resolution. Lab Streaming Layer (Kothe, 2014) was used to synchronize the EMG, finger position, and the experimental program for signal processing. EMG signals were acquired at a sampling rate of 2048 Hz. The electrodes were separated into three groups of A, B, and C. Group A represents flexion muscles located in the inner forearm. Group B represents extensor muscles, which are located in the outer forearm. Group C is optional for the subject who has long arm. The electrodes were separated into two groups of 16-flexion and 16-extension electrodes. The active and passive reference electrodes (CMS and DRL electrodes) placed between the hand and upper arm. However, in subjects that the distance between the hand and upper arm are too far a part for CMS and DRL electrodes to activate, the CMS and DRL electrodes were placed on the back of the subject hand 4 cm apart as shown in Figure 7. This alternative method might increases noise but is unavoidable.

### 2.7. Finger Angle Estimation Model

#### 2.7.1. Musculoskeletal Model

**Figure 8:**
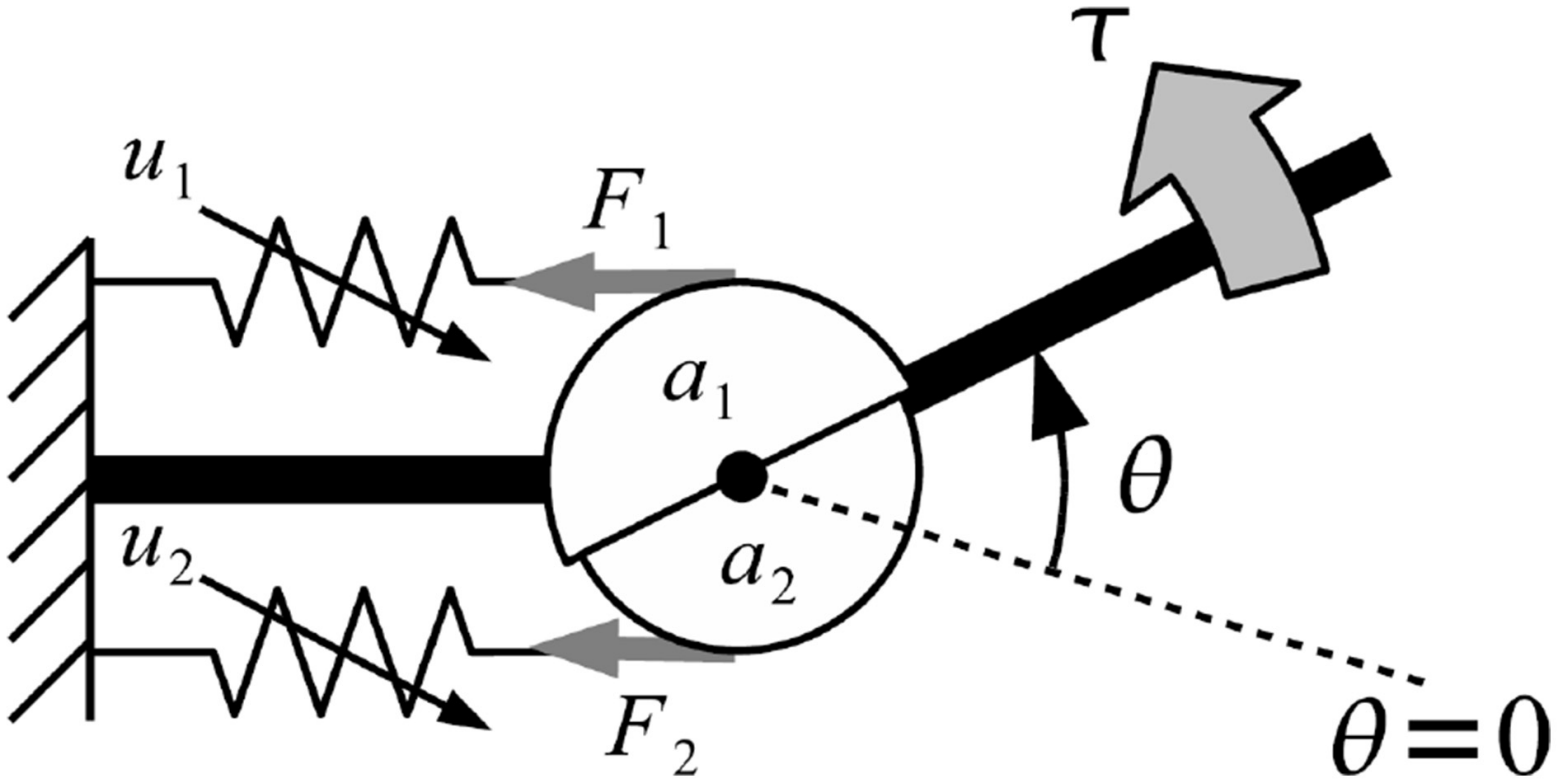
Musculoskeletal model was developed from Mykin model (Shin et al., 2009), represented by flexor and extensor muscles connect to one joint with tendon, torque of muscles (τ_*i*_) represented by muscles contraction (*u*), string constant (*k*_0_ and *k*_1_), and length of muscles (*l*_0_ and *l*_1_).

Musculoskeletal model was developed from Mykin model (Shin et al., 2009), represented by flexor and extensor muscles connect to one joint with tendon, torque of muscles (τ_*i*_) represented by muscles contraction (*u*), string constant (*k*_0_ and *k*_1_), and length of muscles (*l*_0_ and *l*_1_) as shown in Figure 8 (Kawase et al., 2012). Thus, the torque of joint was expressed by sum of all torques from flexor and extensor muscles as shown in Equation (2).

(2) $$\begin{array}{r} \tau_{i}=\overset{2}{\underset{n=1}{\sum }}a_{n}\left(k_{0,n}+k_{1,n}u_{n}\right)\left(l_{0,n}+l_{1,n}u_{n}-a_{n}\theta_{i}\right) \end{array}$$

The parameter τ_*i*_ was torque of finger *i*, *a*_*n*_ denoted moment arm of muscle *n*; *k*_0,*n*_, *k*_1,*n*_, *l*_0,*n*_, *l*_1,*n*_ parameters defined the characteristics of muscle *n* (must be positive value), θ_*i*_ indicated the joint angles of finger *i*, where angle in flexion direction was expressed as positive. Direction indicator of moment arm were expressed as *a*_1_ > 0, *a*_2_ < 0 due to difference in position of the muscles (Kawase et al., 2012).

Equilibrium point was defined as the angle, which torque generated by muscles was balanced to zero and no external force was applied to the joints as shown in Equation (3) (Kawase et al., 2012). Equilibrium point was calculated by solving Equation (2) for the joint angle with condition that τ_*i*_ = 0.

(3) $$\begin{array}{r} \theta_{f,i}^{eq}=\frac{\sum_{n=1}^{2}a_{n}\left(k_{0,n}+k_{1,n}u_{n}\right)\left(l_{0,n}+l_{1,n}u_{n}\right)}{\sum_{n=1}^{2}a_{n}\left(k_{0,n}+k_{1,n}u_{n}\right)} \end{array}$$

The parameter $\theta_{f,i}^{eq}$ was equilibrium point which torque generated by muscles was balanced to zero and no external force was applied to the joints of finger *i*.

Parameter *k*_0,*n*_, *k*_1,*n*_, *l*_0,*n*_, and *l*_1,*n*_ were estimated with a nonlinear regression using 2 sets of training data, (1) data from 5 trials of single finger motion (train) (2) data from 1 trial of mix finger motion (test). In parameter estimation, non-zero values in *a*, *k*_0_, *k*_1_, *l*_0_, and *l*_1_ were calculated, in order to predict finger angle so that it minimize the root-mean-squared errors of the predicted angles.

#### 2.7.2. Linear Regression

LRM is a linear approach to find the relationship between a scalar response and one or more explanatory variables. In this paper, LRM modeled the relation between EMG or IC signal and finger angle as Equation (4).

(4) $$\begin{array}{r} \theta_{f,i}^{li}=\beta_{i,0}+\beta_{i,1}*u_{i,1}+\beta_{i,2}*u_{i,2}+\epsilon \end{array}$$

Where θ_*f,i*_ was finger angle (Thumb, Index, Middle, Ring, Pinky). β_*i*, 0_ was finger angle-intercept. β_*i*, 1_ was regression coefficient for flexor muscles. β_*i*, 2_ was regression coefficient for extensor muscles, *u*_*i*, 1_ was EMG or IC signal from flexor muscles, *u*_*i*, 2_ was EMG or IC signal from extensor muscles, and ϵ was the error term.

β_*i*_ was estimated using MATLAB 2015b (The MathWorks, Inc., U.S.A.), function regress with 2 input and 1 output from train data and 1 test data. After that variable was used to estimate finger angle using the Equation (4) without error term.

### 2.8. Performance Indicators

Due to large number of subjects and trials include in this study, we would like to represent the performance of our proposed system using performance indicators average from all subjects. We select 2 indicators of correlation coefficient (CC) and Root-mean-square-error (RMSE) with mean and standard deviation.

#### 2.8.1. Correlation Coefficient

The correlation coefficient (CC) is a statistic measurement of the strength of relationship between two variables. The number should be between −1.0 and 1.0. Correlation of 1.0 shows a perfect positive correlation (increase and decrease the same amount). A correlation of 0.0 shows no relationship between two variables, and a correlation of −1.0 shows a perfect negative correlation (inverse). The equation represents correlation coefficient was shown in Equation (5).

(5) $$\begin{array}{r} r=\frac{1}{n-1}\overset{n}{\underset{i=1}{\sum }}\left(\frac{x_{i}-\bar{x}}{S_{x}}\right)\left(\frac{y_{i}-ȳ}{S_{y}}\right) \end{array}$$

Where *n* was number of sample, *x* and *y* were two variables, and *S* was standard deviation of each variable set.

The strength of relation based on the value of the correlation coefficient. For example, a value of 0.2 shows weak positive relationship between two variables and likely insignificant. Experts do not consider correlations significant until the value surpasses at least 0.8 and correlation coefficient with an absolute value of 0.9 or higher represent a very strong relationship.

#### 2.8.2. Root-Mean-Square-Error

The root-mean-square-error (RMSE) is a frequently used measurement of the difference between 2 variables. The RMSE represents the square root of the differences between predicted values and observed values. In other words, it represented the concentration of data around the line of best fit. The equation represents root-mean-square-error was shown in Equation (6).

(6) $$\begin{array}{r} RMSE=\sqrt{\frac{\sum_{i=1}^{n}{\left(x_{i}-y_{i}\right)}^{2}}{n}} \end{array}$$

Where *n* was number of sample, *x* and *y* were two variables.

## 3. Results

The flow of signal processing started with the collection of monopole EMG data from the 96 channel Bio-semi sensor. These raw signals required re-reference (using average reference) and band-pass filtering between 5 and 200 Hz to reduce noise (Shaw and Bagha, 2012). The EMG channels for each finger were selected with two criteria: (1) It has to be within the area where we expect the muscle to be located as shown in Figure 5; (2) That channel has the highest correlation between EMG signal and ground-truth finger motion.

The selected EMG channels were fed into AMICA to find the ICs. Using the topological plots, the IC signal that matched with the general anatomical data was selected and used as candidate for the muscle signal. After AMICA, the EMG and IC signals were rectified and low-pass with cut-off frequency about 3 Hz filtered (Koike and Kawato, 1995).

**Figure 9:**
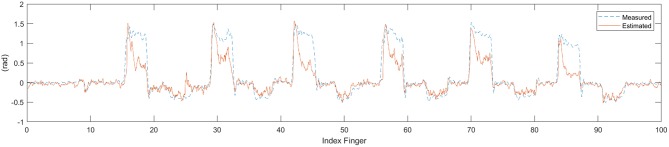
Time series data of estimate finger angle and the ground-truth finger angle from the convolution-pose-machine.

**Figure 10:**
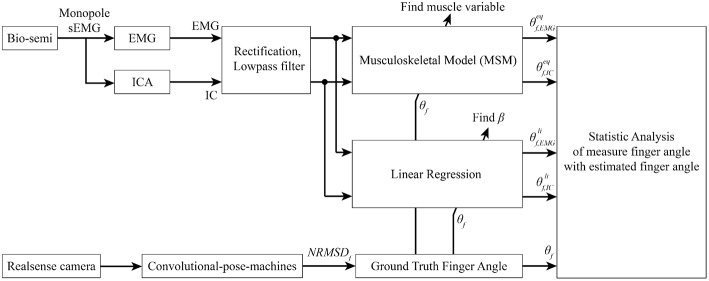
Signal processing flow. Bio-semi sensor collected surface EMG signal from subject and converted to EMG signal using average reference and band pass filter between 5 and 200 Hz. The selected EMG channels were fed into AMICA to find the IC. Both EMG and IC were used to find muscle variables and regression coefficient using ground-truth finger angle from convolutional-pose-machines. Finally, statistical analysis between MSM-EMG, MSM-ICA, LRM-EMG, and LRM-ICA with ground-truth finger angle were performed to confirm the performance of our proposed method.

The EMG and IC signals were fed into the MSM and LRM to obtain estimation of the finger angle from each method (see Figure 9). These estimation were compared with the ground-truth finger angles from the convolution-pose-machine to calculate the CC (see Figure 10). In order to test the hypothesis that the CC value of IC and EMG were significantly different, a paired *t*-test was used. The *p*-values of CC value between IC and EMG were below 0.05 which considered statistically significant.

From now on, statistic analysis result from MSM using EMG and IC signal will be called MSM-EMG and MSM-ICA, respectively. Likewise, statistic analysis result from LRM using EMG and IC signal will be called LRM-EMG and LRM-ICA, respectively.

**Figure 11:**
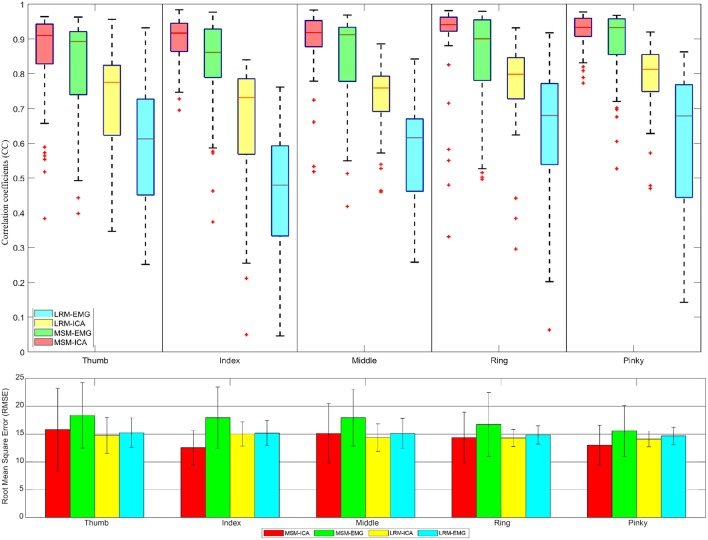
The correlative coefficient value in boxplot manner and root-mean-squre-error in barplot manner of all subjects each performed test experiment (number of data = 50) using variables estimated from train data (5 experiments for each finger) with cross variable. Comparison of four type data between musculoskeletal model (MSM) using ICA (MSM-ICA), musculoskeletal model (MSM) using EMG (MSM-EMG), linear regression model (LRM) using ICA (LRM-ICA), linear regression model (LRM) using EMG (LRM-EMG).

**Figure 12:**
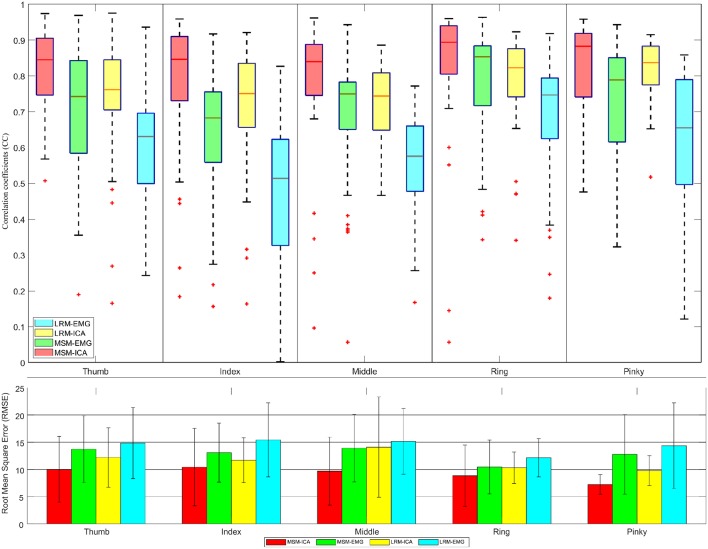
The correlative coefficient value in boxplot manner and root-mean-square-error in barplot manner of all subjects each performed test experiment (number of data = 40) using variables estimated from test data (1 experiments for each finger). Comparison of four type data between musculoskeletal model (MSM) using ICA (MSM-ICA), musculoskeletal model (MSM) using EMG (MSM-EMG), linear regression model (LRM) using ICA (LRM-ICA), linear regression model (LRM) using EMG (LRM-EMG).

Figures 11, 12 showed the CC values in a boxplot and RMSE value in barplot from all subjects for each finger. MSM-ICA was represented in red boxplot with red area, and MSM-EMG was represented in red boxplot with green area. Likewise, LRM-ICA was represented in blue boxplot with red area, and LRM-EMG was represented in blue boxplot with green area. Here, the train data were used to determine the parameters of the MSM, and also to find the ICs. Then, the estimated finger angles were obtained from the test experiment for each of method. The correlation between these estimated finger angles and the ground-truth finger angles were computed (see Figure 11).

The first analysis used train data to find finger muscle variables and regression coefficient in each finger. Then we used test data to estimate finger angle (see Figure 11) and did statistical analysis. The result was CC value comparison between estimated finger angle from MSM, LRM methods and ground-truth finger angle from Realsense with convolutional-pose-machines finger tracking.

The second analysis used one test experiment only to determine finger muscle variables and regression coefficient in each finger. The estimated finger angles from the MSM and LRM were compared with the ground-truth finger angle from the remaining test experiments (see Figure 12). This analysis was done to demonstrate the proposed algorithm's ability to estimate finger angles using a smaller dataset.

## 4. Discussion

Although the EMG signals in combination with MSM and LRM were highly correlated with the ground-truth, the ICA combined with the MSM was even better at estimating the finger angles. This difference between the MSM-ICA and MSM-EMG, LRM-ICA and LRM-EMG is explained by the robustness of the ICA approach to reject noise and motion artifacts in the EMG signal. Table 1 show summary of measured performance of our proposed method, we use two indices of performance for the two estimation methods: correlation coefficients (CC) and the root-mean-squared error (RMSE).

**Table 1:** Average statistic value (10 subjects) of Figures 11, 12.

**Exp**	**Value**	**Thumb**		**Index**		**Middle**		**Ring**		**Pinky**	
		**IC**	**EMG**	**IC**	**EMG**	**IC**	**EMG**	**IC**	**EMG**	**IC**	**EMG**
MSM	CC mean	0.861	0.805	0.936	0.800	0.905	0.850	0.910	0.858	0.941	0.892
train	CC std	0.180	0.169	0.042	0.185	0.086	0.127	0.112	0.147	0.042	0.108
V	RMSE mean	15.81	18.36	12.58	17.95	15.13	17.95	14.38	16.75	13.02	15.56
test	RMSE std	7.430	5.880	3.100	5.540	5.350	5.120	4.570	5.730	3.580	4.570
LRM	CC mean	0.734	0.599	0.662	0.460	0.729	0.568	0.767	0.640	0.785	0.601
train	CC std	0.134	0.183	0.180	0.174	0.106	0.156	0.133	0.190	0.101	0.201
V	RMSE mean	14.75	15.25	15.02	15.19	14.37	15.11	14.30	14.86	14.13	14.68
test	RMSE std	3.222	2.638	2.193	2.241	2.459	2.709	1.548	1.638	1.412	1.579
MSM	CC mean	0.843	0.673	0.803	0.650	0.858	0.681	0.860	0.772	0.907	0.725
test	CC std	0.142	0.229	0.191	0.210	0.103	0.188	0.172	0.189	0.055	0.205
V	RMSE mean	10.01	13.72	10.43	13.09	9.69	13.90	8.85	10.46	7.25	12.77
test	RMSE std	6.037	6.156	7.095	5.425	6.228	6.193	5.642	4.936	1.801	7.299
LRM	CC mean	0.730	0.607	0.709	0.480	0.726	0.554	0.785	0.680	0.815	0.616
test	CC std	0.177	0.170	0.173	0.200	0.112	0.146	0.134	0.183	0.087	0.206
V	RMSE mean	12.20	14.85	11.70	15.43	14.10	15.16	10.32	12.15	9.80	14.36
test	RMSE std	5.477	6.550	4.121	6.785	9.210	6.049	2.884	3.502	2.750	7.852

The first analysis (Figure 11) used test data to find the variable and train model then used such variable and model to estimate finger angle and compared with test data. The objective was to find the performance in an optimal situation that the variable or model was already trained and used for finger angle estimation. This analysis confirmed the performance of MSM-EMG with average CC value at 0.90 ± 0.30 due to high signal to noise ratio of EMG signal itself and clearly distinguishable activation pattern. LRM-EMG performed worst with average CC value at 0.55 ± 0.05 due to the fact that relation between EMG signal and finger angle was not completely linear. Considering RMSE, although MSM-ICA and MSM-EMG showed higher RMSE around averagely 2.0°. The impact of ICA still visible in comparison. As a result, the MSM-ICA and LRM-ICA method was able to provide small increase in performance due to noise reduction and better signal to noise ratio.

The second analysis (Figure 12) used test data from one experiment to find the variable and train model. Then we used such variable and model to estimate finger angle and compared with other test experiment data (do not use in training). This method mimic the actual prosthetic hand control training model as the train data should be minimize while subject might not fully understand the stimulus and move finger randomly. This analysis also confirmed the performance of MSM-EMG when used with low signal to noise ratio with CC value at 0.70 ± 0.10 and LRM-EMG with CC value at 0.55 ± 0.10. In this case, the MSM-ICA and LRM-ICA show significant higher performance and better robustness with CC value at 0.84 ± 0.10 and 0.76 ± 0.06, respectively. Considering RMSE, MSM-ICA shows lower error of 3.0 ± 1.0, 2 ± 1.0, and 4.0 ± 1.0 degree compare to MSM-EMG, LRM-ICA, and LRM-EMG, respectively.

The performance between MSM-ICA and MSM-EMG method showed 10% (CC 0.10) increased in CC values and 3.0−5.0° lower average RMSE. On all fingers, most CC values were higher than 0.7, which indicated a strong uphill linear relationship.

Likewise, the performance of LRM-ICA and LRM-EMG method showed 20% (CC 0.20) improved performance with 1.0–3.0 degree lower average RMSE.

The difference between ICA and EMG methods indicated that the ICA method can reduce noise and increase the signal to noise ratio for the MSM model with high reliability. However the ICA method needs human supervision, as the motion artifact of muscles are higher when size of muscles increased.

## 5. Conclusions

In this research, we proposed new method to replace conventional EMG sensor placement with array EMG system. The array EMG system covered forearm by Bio-semi sensor and detected the signal simultaneously. The signal source can be determined after the experiment. And the IC can be analyzed to find better source of signal. The setup time of this system is about 30 min with two operators. There is no need to find the location of muscles which is advantageous in case of the multiple number of muscles or unknown location. In post experimental analysis, the problem of muscle movement under the skin do not affect the signal acquisition as in the conventional EMG sensor. Also the proposed method provided overall better signal to noise ratio and was able to use ICA to estimate inside muscles activity (see Table 2).

**Table 2:** Array EMG system compare to conventional EMG sensor.

	**Array EMG system**	**EMG sensor**
Do not need information of muscles location	✓	✗
Do not need predefined number of muscles	✓	✗
Can handle muscles location changes during experiment	Post analysis adjustment	✗
Use ICA to reduce noise	✓	✗
Signal to noise ratio	3.0–100.0	1.2–4.5

The actual finger angle was calculated from the NRMSE of convolutional-pose-machines-tensorflow with the trigger to determine the direction of finger motion. The EMG signal from flexor and extensor muscles were determined by CC value between finger motion and EMG signal. IC was extracted from all EMG signals using AMICA (Palmer et al., 2012). The EMG and IC signals that most fitting flexion period were considered the EMG and IC signal of flexor muscles. Likewise, the EMG and IC signals that most fitting extension period were considered the EMG and IC signal of extensor muscles. The performance of our method showed relatively higher CC value using MSM-ICA and LRM-ICA to estimate finger angle. For practical application in robotic hand, we designed the experiment so that all fingers move in flexion and extension manners continuously without interruption. This experiment showed performance according to the subject within the acceptable range of CC value: 0.7 ± 0.2. In the case of prosthetic hand control for amputee subject, the muscle locations can be differ from general anatomy data and result in difficulty to find the best location for finger muscles. The proposed system provided a bypass method to collect the data from the entire forearm and estimated the location of muscles.

The advantages of our system over conventional EMG system are:

- Standard protocol for all subjects participate in the experiment (Reduce complexity).
- Reduce time used for select location of EMG sensor due to anatomy variation of each subject.
- Able to detect deep muscle (such as finger muscles) EMG signal using multiple sensors and signal processing.
- Able to compensate for muscle movement under skin that generate disruption of EMG signal caused by adjacent joint movement.
- Higher overall performance using the same regression method.
- Do not limit number of muscles.

And the disadvantages are:

- Require at least 30 min before experiment to attach sensors.
- Target area should not move during experiment.

In the future, we would like to combine many sensor signals into two groups of single joint motion by developing the MSM model to be able to use multiple muscles to estimate joint angle. The machine learning and deep learning also able to utilize Array EMG system better than normal linear regression.

## Data Availability

The datasets analyzed in this manuscript are not publicly available. Requests to access the datasets should be directed to stapornchaisit.s.aa@m.titech.ac.jp, koike@pi.titech.ac.jp.

## Ethics Statement

This studies involving human participants were reviewed and approved by Tokyo Institute of Technology and carried out in accordance with the Declaration of Helsinki. The patients/participants provided their written informed consent to participate in this study.

## Author Contributions

NY and YKo developed the concept. SS, YKo, and NY developed the experimental design. SS, YKi, and NY collected the data and analyzed the data. SS, AT, NY, and YKo wrote the paper.

### Conflict of Interest Statement

The authors declare that the research was conducted in the absence of any commercial or financial relationships that could be construed as a potential conflict of interest.
